# Immunohistochemical Expression of Programmed Death Ligand 1 in Oral Extranodal Diffuse Large B Cell Lymphoma

**DOI:** 10.1055/s-0042-1747951

**Published:** 2022-08-09

**Authors:** Rania Hanafi Mahmoud Said, Fatma F. Hussein, Amal M. El-Deeb

**Affiliations:** 1 Department of Oral Pathology, Faculty of Dentistry, Suez Canal University, El Salam District Ismailia Governorate, Egypt; 2 Department of Oral Pathology in Faculty of Dentistry, Umm Al Qura University, Kingdom of Saudi Arabia; 3 Department of Oral Medicine, Oral Diagnosis and Periodontology, Faculty of Dentistry, Minia University, Minya, Menia Governorate, Egypt; 4 Faculty of Dentistry, Umm Al Qura University, Kingdom of Saudi Arabia; 5 Department of Oral Pathology, Faculty of Dentistry, Tanta University, Tanta, Gharbia Governorate, Egypt

**Keywords:** oral extranodal diffuse B cell lymphoma, diffuse large B cell lymphoma, programmed cell death ligand 1

## Abstract

**Objective** Lymphomas are the third most common cancer after squamous cell carcinoma and salivary gland tumors. Extranodal diffuse B cell lymphoma (DBCL) represents 30 to 58% of non-Hodgkin's lymphoma. One of the major problems of DBCL is the high likelihood of disease relapse following treatment. A recent trend in the treatment of diffuse large B cell lymphoma (DLBCL) is blockage of an immune checkpoint inhibitor that targets the programmed death of cell ligand 1 receptors (PD-L1). PD-L1 activation results in negative regulatory signals that induce apoptosis and inhibit tumor antigen-specific T cells allowing immune evasion of the tumor.

The aim of this aim is to measure the expression level of PD-L1 on oral tissue samples from DLBCL patients using immunohistochemistry.

**Materials and Methods** This current study was performed at the Faculty of Dentistry, Tanta University, Egypt. Ethical approval was conducted from Faculty of Dentistry, Tanta University. Tissue samples were collected from 13 patients diagnosed with oral extranodal DLBCL) nongerminal center B cell like subtype. Both hematoxylin and eosin and immunohistochemical staining (The avidin-biotin-complex procedure) was performed with anti-PD-L1 antibody (clone number: 28–8, Abcam, Cambridge, Massachusetts, United States).

Cytoplasmic and/or membranous positive intensity was graded as follows: very mild staining, mild staining, moderate staining, and intense staining using Image J, 1.41a (National Institutes of Health, United States) image analysis software. The mean area fraction of the stained cells was calculated by counting immunostained cells in three fields of each case by two pathologists. Data was entered in SPSS program for analysis.

**Results** PD-L1 was overexpressed on tumor cells of oral extranodal DLBCL than control cells from lesion free areas of oral tissues of the same patient.

## Introduction

Oral cavity lymphomas are the third most common malignant lesions after squamous cell carcinoma and salivary gland tumors. 1 Lymphoma is a heterogeneous group of hematological neoplasms characterized by proliferation of malignant lymphoid cells, or their precursors, and have been classified into non-Hodgkin's lymphomas (NHLs) (70–80%) and Hodgkin that represent 20 to 30% of cases. 2 Among all the NHL subtypes arising in the oral cavity and the jaw bones, diffuse large B cell lymphoma (DLBCL) represents the most known lymphoid neoplasm, which represents 30 to 58% of NHL. DLBCLs emerge either from lymph nodes or from extranodal sites. 3 Differences in medical and family history, lifestyle, predisposing factors, natural history, clinical presentation, and molecular pathogenesis of patients indicate that extranodal DLBCLs have distinct contributing factors. 3 4 Among all head and neck NHLs, ∼55.5% of them are extranodal lymphomas, while the remaining 44.5% are nodal forms. 5

Extranodal DLBLs originate from every anatomic site such as gastrointestinal tract (GIT) (most common), head and neck, skin, central nervous system, bone, testis, breast, pancreas, rarely adrenal, and the genitourinary tract. 6 In head and neck, after GIT, DLBCLs are the most frequent, and have been categorized as the second most probable known site of extranodal lymphomas, 7 8 where DLBCLs frequently take place in the ring of Waldeyer, paranasal sinuses, orbit, thyroid glands, and salivary glands. 9 In terms of the oral cavity, less than 5% of oral malignant disease, which commonly develops in submucosal tissues of gingiva, tongue, and palate, and sometimes rises as swelling, pain, ulceration, tooth mobility, or bone destruction, is represented by the extranodal lymphoma. 10

For DLBCL patients, the prognosis is heterogeneous and differs among patients having similar pathologic types, 11 12 despite the fact that the current standard chemotherapy regimen boosts the rates of response and results in improved patient survival. However, ∼43% of patients fail to respond or display relapse or chemoresistance. For those reasons, developing new prognostic biomarkers can be effectively used to not only classify and categorize DLBCL in accordance with severity and prognosis but also to serve as therapeutic targets to prolong patient survival. 13 DLBCL physiopathology is dependent on both the tumor cells and the microenvironment (ME) of DLBCL, which is key for its carcinogenesis. In the ME, the stromal cells of the tumor and the immune infiltrate composition have an effect on DLBCL progression. 14 15 16 One of the recent trends in the treatment of DLBCL is blockade of an immune checkpoint that targets the programmed death of cell ligand 1 receptors (PD-L1). 17 These checkpoints are receptors found on the surface of immune cells such as B-lymphocytes, T lymphocytes, dendritic cells and macrophages, and are very important in playing essential roles in tumor progression. 18

PD-L1 is also known as differentiation 274 cluster (CD274), an important B7 family member. 19 Being an inhibitory ligand, the PD-L1 represents an essential immune checkpoint that has key roles to play in regulating cellular, adaptive, and humoral immune responses. 20 PD-L1 binds to programmed death of cell protein 1 (PD-1) receptor, which transmits negative regulatory signals to bolster tumor immune evasion and stimulate tumor antigen-specific T cells' apoptosis and immune incompetence. Furthermore, the cell-intrinsic signaling of PD-L1 preserves tumor cells from interferon (IFN) cytotoxicity and hastens the progression of the tumor. 21 Therefore, a vital role is played by PD-1/PD-L1 pathway in the peripheral tolerance. 22 In addition, it mediates the inhibitory signals disclosing antitumor immunity. Aberrant expression of PD-L1 has been shown to have an association with the undesirable prognosis of several kinds of cancers. 20 21 Several studies have reported the upregulated expression of PD-L1 in lymphoma and illustrated its association with the prognosis of DLBCL 23 24 however, the prognostic role of PD-L1 expression in DLBCL remains unclear.

## Research Aims

The aim of this study was to evaluate the immunohistochemical expression of the PD-L1 in oral extranodal DLBCL.

## Materials and Methods

**Fig. 1:**
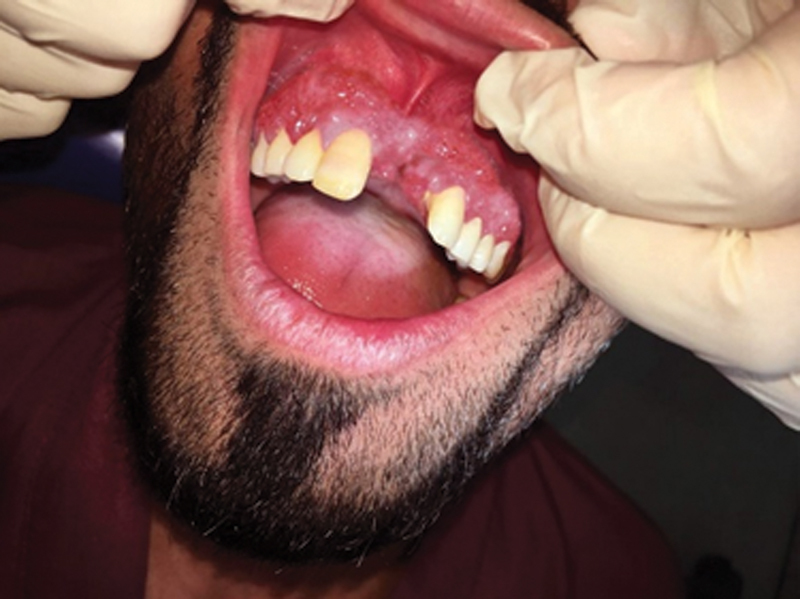
Maxillary gingival enlargement induced by diffuse large B-cell lymphoma infiltration.

**Fig. 2:**
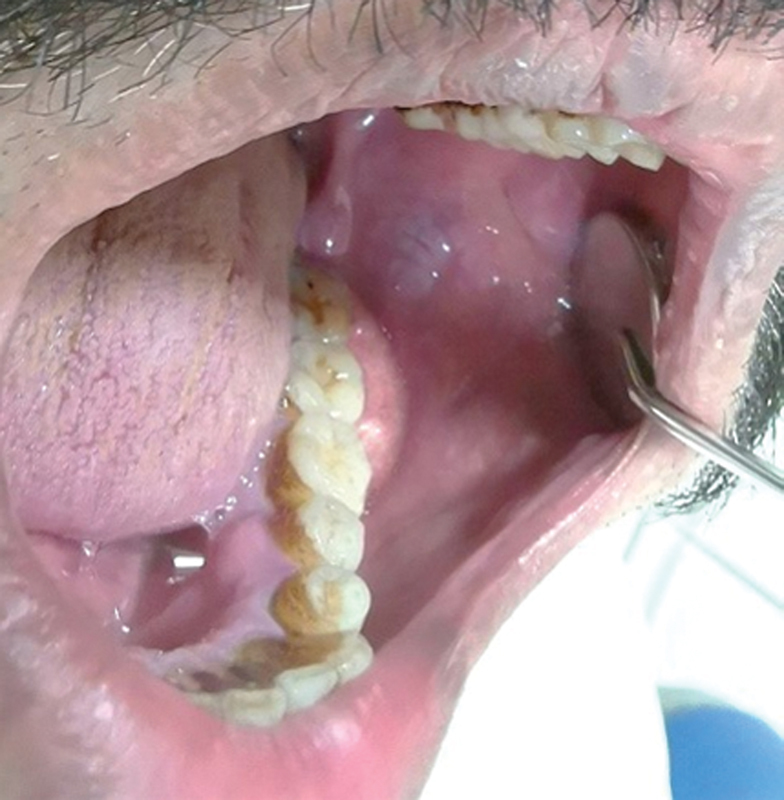
Large mass involving gingival tissue of left second and third mandibular molars and extending to obliterate mucobuccal fold.

Consent was obtained from 13 patients (9 females and 4 males) with oral extranodal DLBCL nongerminal center B cell like (non-GCB subtype). Patients' ages ranged between 35 and 64. Two biopsies were taken from each patient. The thirteen biopsies were divided as ten cases from gingiva and three others from palate ( Figs. 1 and 2 ). All the oral biopsies were divided into two groups. Tumor group (group T) containing biopsies that were taken from tumor sites, and control group (group C) containing biopsies taken from lesion free areas of oral tissues of the same patient. As the onset of DLBCL was correlated to infection of some viruses including hepatitis C virus (HCV) and human immunodeficiency virus, patients were confirmed negative for acquired immunodeficiency syndrome and HCV and had no history of receiving immunotherapy. Specimens were collected and immersed in 10% formalin for 24 to 48 hours before they were washed in phosphate-buffered saline and then embedded in paraffin. The embedding process was performed by three immersions in 70, 80, and 96% ethanol (90 minutes each); three immersions in absolute ethanol (60 minutes each); two immersions in xylol (90 minutes each); and two immersions in liquid paraffin at 60°C (120 minutes each). The avidin–biotin complex procedure was performed with anti-PD-L1 antibody (clone number: 28–8, Abcam, Cambridge, Massachusetts, United States) that was applied at a 1:200 dilution.

i. 4μm sections were obtained and applied to clean glass slides and stained with hematoxylin and eosin for examination under light microscope.

From each paraffin block, three sections (5 µm in thickness) were mounted on positively charged slides. They were applied to clean glass slides and stained with streptavidin-biotin immunohistochemical method for PD-L1 antibody. All stained slides were examined with a multihead microscope. The intensity of cytoplasmic and/or membranous positivity was described as either very mild staining, mild staining, moderate staining, and intense staining. The histomorphometric analysis was performed using Image J, 1.41a, (National Institutes of Health, United States) image analysis software. 25

All images were captured using digital camera (2951 Ishikawa-machi, Hachioji-shi, Tokyo 192-8507, Japan) mounted on a light microscope (BX60, Olympus, Japan). Images were then transferred to the computer system, for analysis in the Precision Measurement Unit, Biotechnology Department, Faculty of Science, Tanta University.

ii. Three fields from each slide were counted by two pathologists from Oral Pathology Department, Faculty of Dentistry, Tanta University and Suez Canal university. PD-L1 samples were considered positively expressed when ≥ 5% of counted tumor cells were stained with anti-PD-L1 (either membranous and/or cytoplasmic). The mean area fraction (MAF) for each case was then calculated by addition of the area fractions of the three fields and dividing the result by three. The total of MAF was then calculated and used for statistical analysis. 20

### Statistical Analysis

Data was tabulated and displayed as MAF and standard deviations then analyzed using SPSS version 20. Unpaired *t* -test was done to compare between the MAF differences of both groups. The *p* -value was considered significant if its value was less than or equal to ≤ 0.005. Pearson test was used to correlate between immunoexpression of anti-PD-L1 in tumor areas and the age of patients.

## Results

**Fig. 3:**
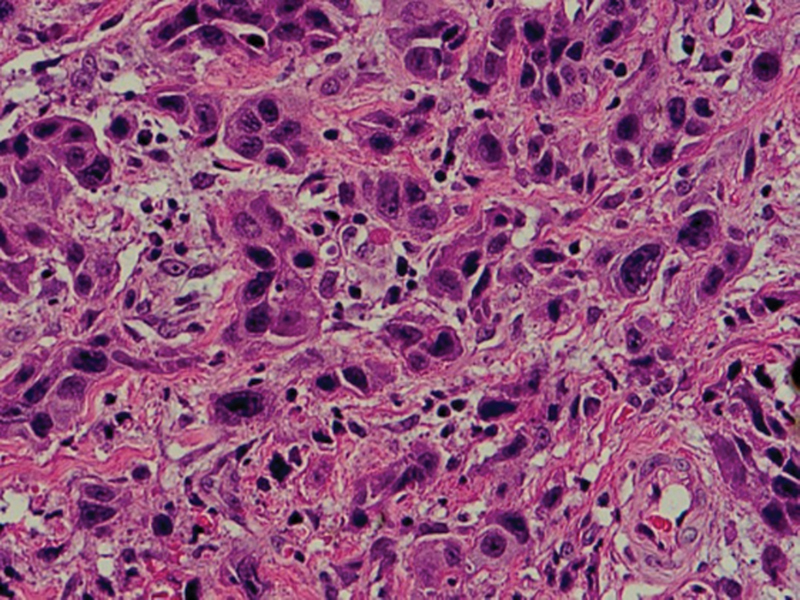
A photomicrograph of extranodal diffuse large B-cell lymphoma showing aggregations of proliferated large B cells with rounded, oval, irregular nuclei (may be lobulated), distinct nucleoli, and scanty cytoplasm (hematoxylin and eosin Orig.mag.X20).

**Fig. 4:**
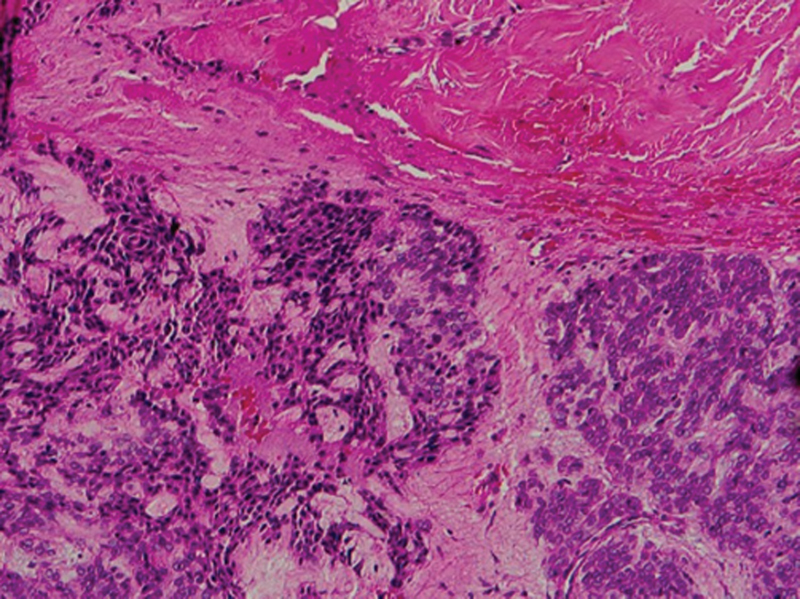
Photomicrographs showing extranodal diffuse large B-cell lymphoma aggregations of proliferated large B cells with pleomorphism and hyperchromatism. The cells show rounded, oval, irregular nuclei (may be lobulated), distinct nucleoli, and scanty cytoplasm.

**Fig. 5:**
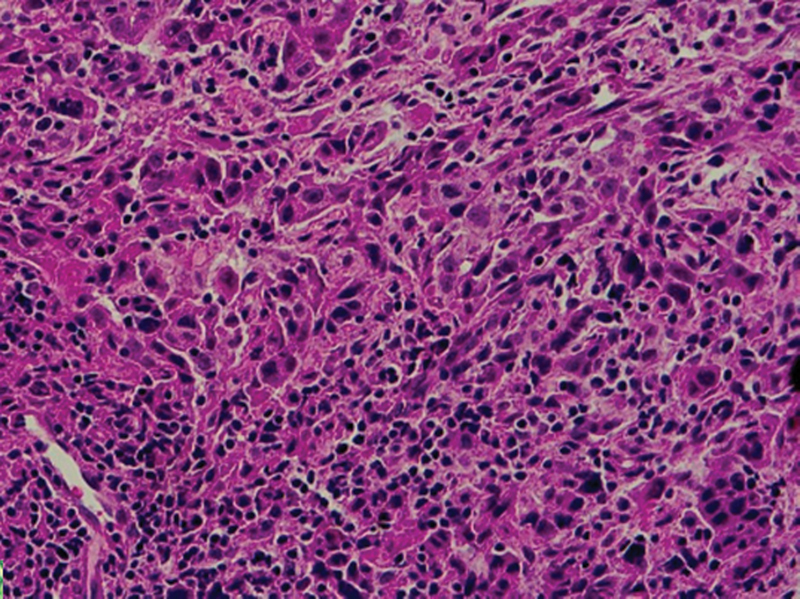
Photomicrographs showing extranodal diffuse large B-cell lymphoma aggregations of proliferated large B cells with pleomorphism and hyperchromatism. The cells show rounded, oval, irregular nuclei (may be lobulated), distinct nucleoli, and scanty cytoplasm ( Fig. 4 hematoxylin and eosin Orig.mag.X10 and Fig. 5 Orig.mag.X20).

Light microscopic examination shows aggregations of extranodal proliferated large B cells with rounded, oval, irregular nuclei (may be lobulated), distinct nucleoli, and scanty cytoplasm ( Figs. 3 , 4 , 5 ).

### Immunohistochemical Staining

**Fig. 6:**
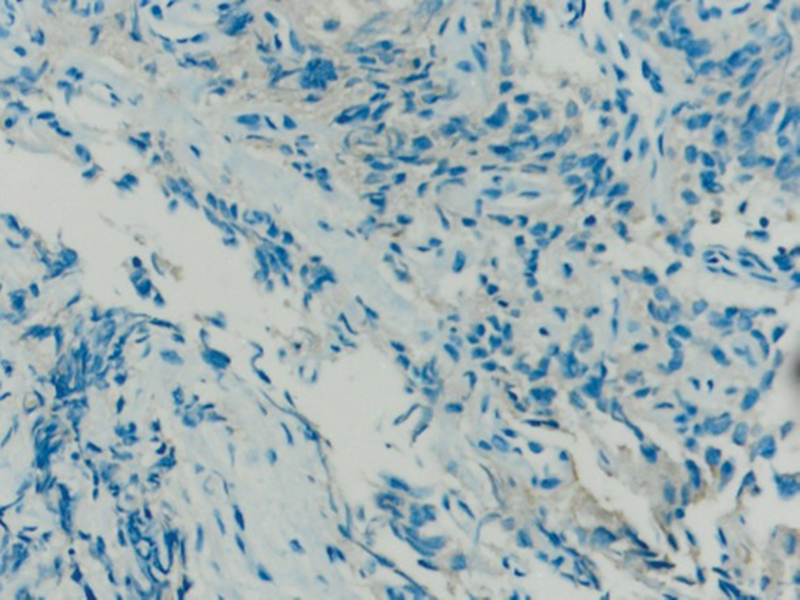
A photomicrograph showing very mild immunoexpression of programmed death of cell ligand 1 (PD-L1) in group C (PD-L1 antibody Orig.mag. X10).

Immunohistochemical expression of anti-PD-L1 antibody shows very mild immunoexpression in cells taken from control biopsies (group C) ( Fig. 6 ). The staining appears on the cytoplasmic cell membranes of T-lymphocytes, B-lymphocytes, and macrophages.

**Fig. 7:**
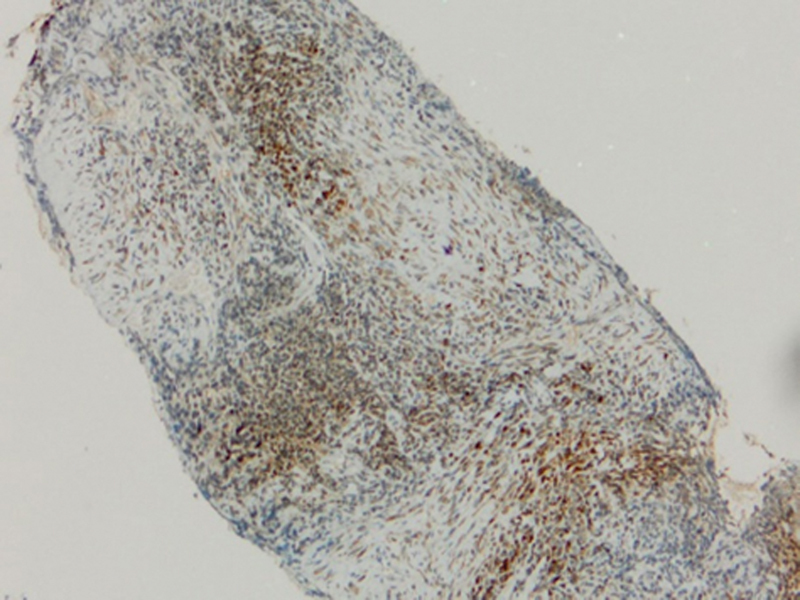
A photomicrograph showing mild cytoplasmic membrane staining for programmed death of cell ligand 1 (PD-L1) in group T (PD-L1 antibody Orig.mag.X10).

**Fig. 8:**
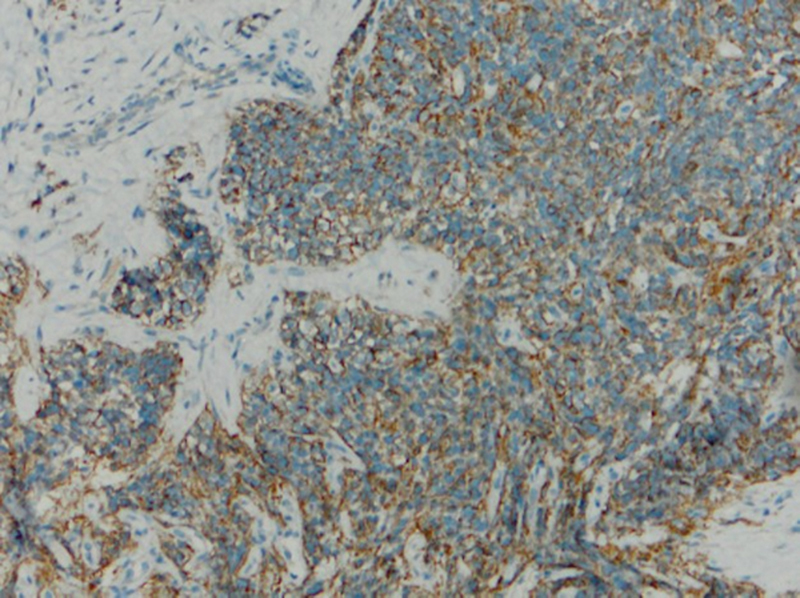
A photomicrograph showing mild cytoplasmic membrane staining for programmed death of cell ligand 1 (PD-L1) in group T (PD-L1 antibody Orig.mag.X10).

**Fig. 9:**
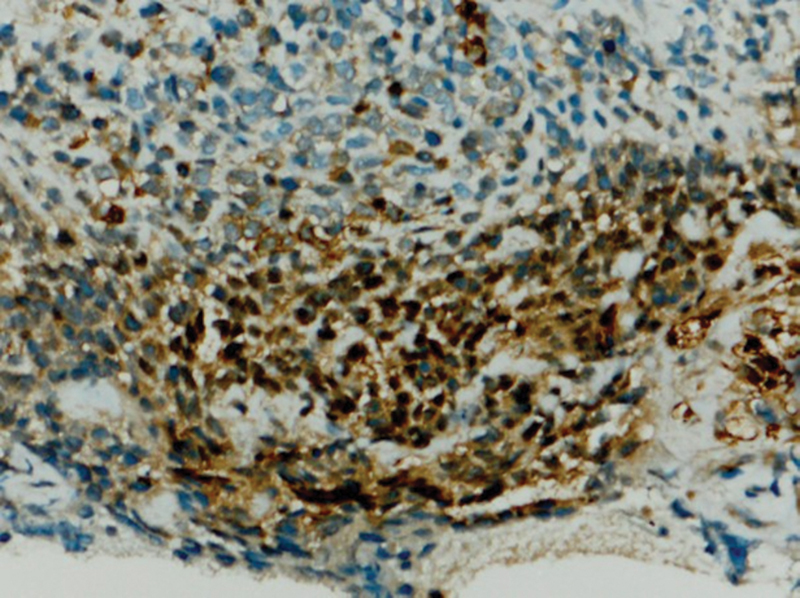
A photomicrograph showing moderate cytoplasmic membrane that stains for programmed death of cell ligand 1 (PD-L1) in group T (PD-L1 antibody Orig.mag.X20).

**Fig. 10:**
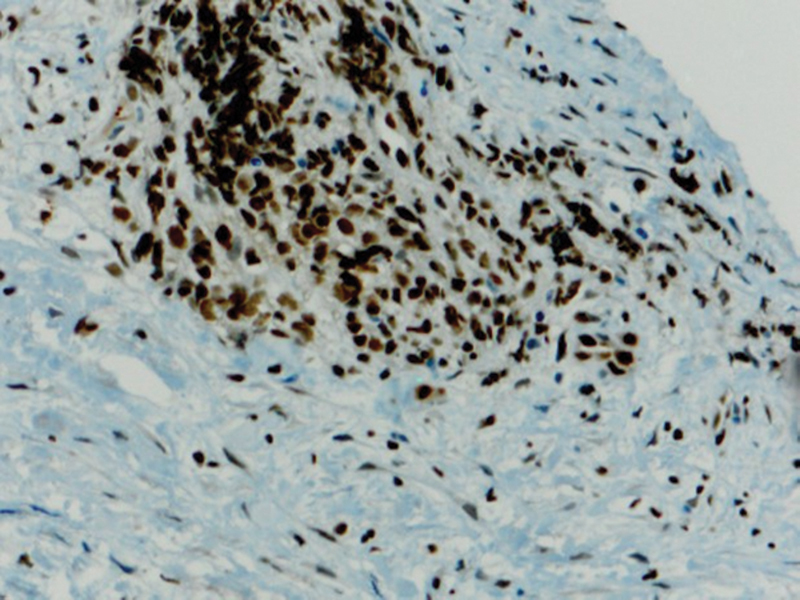
A photomicrograph showing intense cytoplasmic membrane staining for programmed death of cell ligand 1 (PD-L1) in group T (PD-L1 antibody Orig.mag.X20).

**Fig. 11:**
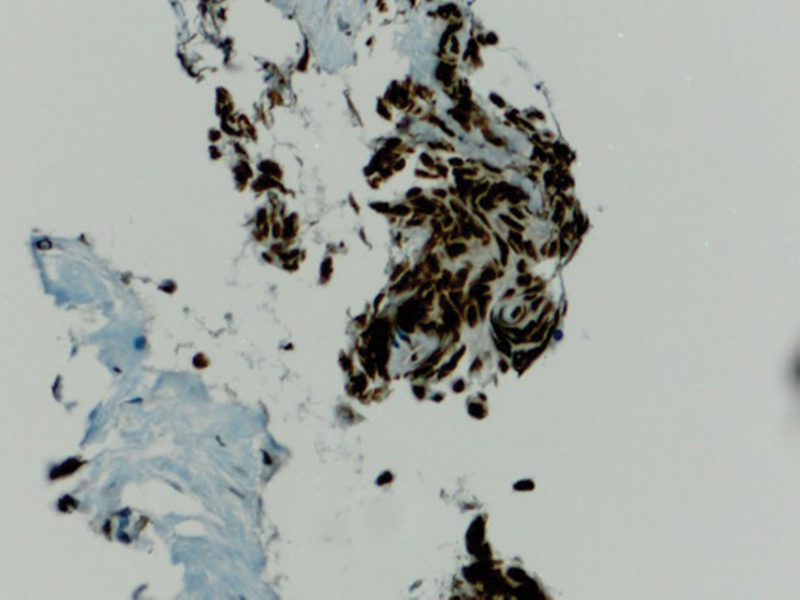
A photomicrograph showing intense cytoplasmic membrane that stains for programmed death of cell ligand 1 (PD-L1) in group T (PD-L1 antibody Orig.mag.X20).

The staining appears mild ( Figs. 7 and 8 ), moderate ( Figs. 9 and 10 ), and intense cytoplasmic staining in different tumor cells (group T) ( Fig. 11 ).

**Fig. 12:**
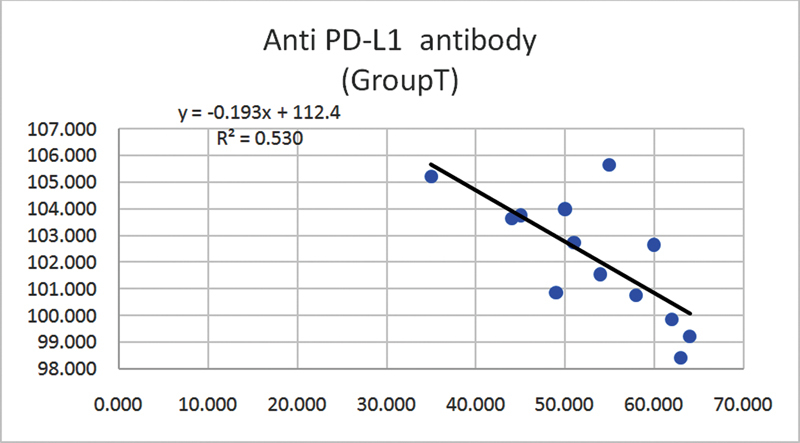
Correlation between immunoexpression between the expression of anti-programmed death of cell ligand 1 (PD-L1) in tumor areas and the age of patients (Pearson test).

Nine of the biopsies were female biopsies (69%) versus four male biopsies (31%). The age of patients ranged from 35 to 64 ( Fig. 12 ). The anti-PD-L1 immunoexpression was obvious in group T than in group C ( Table 1 ). There is a significant difference between the immunoexpression of anti-PD-L1 in group C and the immunoexpression of anti-PD-L1 in group T ( *p* -value = 0.00012) ( Table 2 ).

**Table 1:** The relationship between the MAF of anti PD-L1 immunoexpression in group C (control Group), and the expression of anti PD-L1 in group T (tumor areas)

Sex	Age	Group C	Group T
M	44	72.999	103.654
M	55	70.616	105.656
M	60	68.249	102.667
M	58	75.885	100.776
F	45	73.634	103.765
F	62	77.124	99.872
F	50	72.799	104.001
F	54	70.616	101.554
F	49	68.249	100.878
F	64	75.885	99.234
F	63	73.634	98.432
F	51	77.124	102.743
F	35	70.645	105.225

Abbreviations: MAF, mean area fraction; PD-L1, programmed death of cell ligand 1.

**Table 2:** Comparison between the MAF of anti PD-L1 immunoexpression in group C (control group) and group T (tumor group)

Group C		Group T		Results	
Average	STD	Average	STD	*t* -test	*p* -Value
72.88146	3.07381	102.18900	2.27340	0.00000	0.00012

Abbreviations: MAF, mean area fraction; PD-L1, programmed death of cell ligand 1.

## Discussion

The findings of this study reveal that the mean age of the DBCL patients is 51.1 and the majority of patients were females. This result was close to a Malaysian study by Ramanathan et al; on 40 patients with oral extranodal DBCL, the median age of patients was 47 but the majority were males. 26 In another study by van der Waal et al, the mean age of patients was 59. 27 In a study by Shah et al, the mean of patients age was 42.6. 28 In a study by Kemp et al,, 53% of oral extranodal DBCLs were in females. 29 However, in all mentioned studies, the number of patients in samples did not exceed 42 and did not contain a wide variety of ethnic backgrounds (Egyptians, Indians, Chinese, and Malaysian).

In the present study, we note the majority of cases were in gingiva and palate that are consistent with the results of Shat et al 28 and Takahashi et al. 30 The palate and gingiva were described as the common sites of oral extranodal DBCL and salivary glands are the second common sites 31 with the maxilla and mandible are the rare sites as described by Eisenbud et al, they found out of 31 cases of oral NHL only 14 patients with bony involvement, five were in the mandible. 32

In this study, immunoexpression of anti-PD-L1 was found to be significantly higher in the tumor group compared with the control group. Results are consistent with the study of Menter et al that reported overexpression of PD-L1 on cells of Hodgkin and NHL cells and blood lymphocytes. They correlated the overexpression of PD-L1 in DLBCLs with the tumor prognosis. 33

In normal physiologic conditions, PDL-1 expression on normal human tissues was confined to cells of the tonsils, placenta, 34 and some macrophage like cells in liver and lung. 35 Tumor ME causes PD-L1 to be overexpressed on tumor cells. Tumor existence leads to increase the inflammatory cytokines that leads to tremendous release of interferon (IFN-γ) that was initially released to protect tissues from damage by released cytokines. IFN-γ results in overexpressing the PD-L1 on tumor cells. PD-L1 overexpression on tumor cells prevents T cell activation and causes T cell exhaustion instead of causing subsequent apoptosis. 36

Overexpression of PD-L1 in oral extranodal DLBCLs was found to be multifactorial. In 20% of DLBCLs cases, PD-L1 overexpression on tumor cells' surfaces refers to genetic alterations. 37 Another factor that may cause PDL-1 overexpression on tumor cells is Epstein–Barr virus initiation (EBV) activation, which may drive immune tolerance. 38 Many studies correlate increased EBV infection with the development of aggressive B cell lymphoma. 39 The most acceptable scenario is the correlation of increased IFN-γ in inflammatory process that accompanied the tumor progression as explained above.

Immunohistochemistry results showed very mild immunoexpression of anti-PD-L1 in control groups. These results strengthen the hypothesis that correlates PDL-1 overexpression of PD-L1 on tumor cells with genetic alterations or the involvement of EBV infection. 37 39 In some studies, overexpression of PD-L1 was measured on blood cells and was taken as control beside the solid tumor cells. 40

## Conclusion

We show that PDL-1 activation is increased on oral tumor cells of patients with DLBCLs. Further studies are needed to understand the mechanism of action.

## References

[1] Silva T D Ferreira C B Leite G B de Menezes Pontes J R Antunes H S Oral manifestations of lymphoma: a systematic review Ecancermedicalscience 2016 10 665 27594910 10.3332/ecancer.2016.665 PMC4990057

[2] Boussios S Zerdes I Vassou A Extranodal diffuse large B-cell lymphomas: a retrospective case series and review of the literature Hematol Rep 2018 10 01 7070 29721249 10.4081/hr.2018.7070 PMC5907641

[3] Møller M B Pedersen N T Christensen B E Diffuse large B-cell lymphoma: clinical implications of extranodal versus nodal presentation–a population-based study of 1575 cases Br J Haematol 2004 124 02 151 159 14687024 10.1046/j.1365-2141.2003.04749.x

[4] ESMO Guidelines Committee Vitolo U Seymour J F Martelli M Extranodal diffuse large B-cell lymphoma (DLBCL) and primary mediastinal B-cell lymphoma: ESMO Clinical Practice Guidelines for diagnosis, treatment and follow-up Ann Oncol 2016 27 05 v91 v102 27377716 10.1093/annonc/mdw175

[5] Iguchi H Wada T Matsushita N Oishi M Yamane H Anatomic distribution of hematolymphoid malignancies in the head and neck: 7 years of experience with 122 patients in a single institution Acta Otolaryngol 2012 132 11 1224 1231 23025415 10.3109/00016489.2012.694474

[6] Bangash M Hussain I Zakaria M Piracha M Pattern of extranodal involvement in non-Hodgkin's lymphoma Pak Armed Forces Med J 2014 64 04 605 608

[7] Hart S Horsman J M Radstone C R Hancock H Goepel J R Hancock B W Localised extranodal lymphoma of the head and neck: the Sheffield Lymphoma Group experience (1971-2000) Clin Oncol (R Coll Radiol) 2004 16 03 186 192 15191005 10.1016/j.clon.2003.10.010

[8] Bagan J V Carbonell F Gómez M J Extra-nodal B-cell non-Hodgkin's lymphomas of the head and neck: a study of 68 cases Am J Otolaryngol 2015 36 01 57 62 25456517 10.1016/j.amjoto.2014.10.008

[9] Gotoa M Saitob M Kuroyanagic N Intraosseous lymphoma of the oral and maxillofacial regions: report of our experiences, involving some difficult cases to be diagnosed, case report J Oral Maxillofac Surg Med Pathol 2016 28 41 46

[10] Zapater E Bagán J V Carbonell F Basterra J Malignant lymphoma of the head and neck Oral Dis 2010 16 02 119 128 20374502 10.1111/j.1601-0825.2009.01586.x

[11] Chi H S Lee K W Chiang F Y Head and neck extranodal lymphoma in a single institute: a 17-year retrospective analysis Kaohsiung J Med Sci 2012 28 08 435 441 22892165 10.1016/j.kjms.2012.02.014 PMC11916381

[12] Roschewski M Staudt L M Wilson W H Diffuse large B-cell lymphoma-treatment approaches in the molecular era Nat Rev Clin Oncol 2014 11 01 12 23 24217204 10.1038/nrclinonc.2013.197 PMC7709161

[13] Liu J Quan L Zhang C Liu A Tong D Wang J Over-activated PD-1/PD-L1 axis facilitates the chemoresistance of diffuse large B-cell lymphoma cells to the CHOP regimen Oncol Lett 2018 15 03 3321 3328 29435074 10.3892/ol.2017.7682 PMC5778856

[14] Keane C Gill D Vari F Cross D Griffiths L Gandhi M CD4(+) tumor infiltrating lymphocytes are prognostic and independent of R-IPI in patients with DLBCL receiving R-CHOP chemo-immunotherapy Am J Hematol 2013 88 04 273 276 23460351 10.1002/ajh.23398

[15] Keane C Vari F Hertzberg M Ratios of T-cell immune effectors and checkpoint molecules as prognostic biomarkers in diffuse large B-cell lymphoma: a population-based study Lancet Haematol 2015 2 10 e445 e455 26686046 10.1016/S2352-3026(15)00150-7

[16] Kridel R Steidl C Gascoyne R D Tumor-associated macrophages in diffuse large B-cell lymphoma Haematologica 2015 100 02 143 145 25638802 10.3324/haematol.2015.124008 PMC4803134

[17] Bachy E Coiffier B Anti-PD1 antibody: a new approach to treatment of lymphomas Lancet Oncol 2014 15 01 7 8 24332517 10.1016/S1470-2045(13)70587-4

[18] Kim J R Moon Y J Kwon K S Tumor infiltrating PD1-positive lymphocytes and the expression of PD-L1 predict poor prognosis of soft tissue sarcomas PLoS One 2013 8 12 e82870 24349382 10.1371/journal.pone.0082870 PMC3859621

[19] Zhao S Zhang M Zhanget Y The prognostic value of programmed cell death ligand 1 expression in non-Hodgkin lymphoma: a meta-analysis Cancer Biol Med 2018 15 03 290 298 30197796 10.20892/j.issn.2095-3941.2018.0047 PMC6121049

[20] Hu L Y Xu X L Rao H L Expression and clinical value of programmed cell death-ligand 1 (PD-L1) in diffuse large B cell lymphoma: a retrospective study Chin J Cancer 2017 36 01 94 29246182 10.1186/s40880-017-0262-z PMC5732416

[21] Gato-Cañas M Zuazo M Arasanz H PDL1 Signals through conserved sequence motifs to overcome interferon-mediated cytotoxicity Cell Rep 2017 20 08 1818 1829 28834746 10.1016/j.celrep.2017.07.075

[22] Keir M E Butte M J Freeman G J Sharpe A H PD-1 and its ligands in tolerance and immunity Annu Rev Immunol 2008 26 677 704 18173375 10.1146/annurev.immunol.26.021607.090331 PMC10637733

[23] Westin J R Chu F Zhang M Safety and activity of PD1 blockade by pidilizumab in combination with rituximab in patients with relapsed follicular lymphoma: a single group, open-label, phase 2 trial Lancet Oncol 2014 15 01 69 77 24332512 10.1016/S1470-2045(13)70551-5 PMC3922714

[24] Kiyasu J Miyoshi H Hirata A Expression of programmed cell death ligand 1 is associated with poor overall survival in patients with diffuse large B-cell lymphoma Blood 2015 126 19 2193 2201 26239088 10.1182/blood-2015-02-629600 PMC4635115

[25] Kaczmarek E Górna A Majewski P Techniques of image analysis for quantitative immunohistochemistry Rocz Akad Med Bialymst 2004 49(1, Suppl 1):155 158 15638406

[26] Ramanathan A Mahmoud H A Hui L P Mei N Y Valliappan V Zain R B Oral extranodal non-Hodgkin's lymphoma: series of forty two cases in Malaysia Asian Pac J Cancer Prev 2014 15 04 1633 1637 24641380 10.7314/apjcp.2014.15.4.1633

[27] van der Waal R I Huijgens P C van der Valk P van der Waal I Characteristics of 40 primary extranodal non-Hodgkin lymphomas of the oral cavity in perspective of the new WHO classification and the International Prognostic Index Int J Oral Maxillofac Surg 2005 34 04 391 395 16053848 10.1016/j.ijom.2004.08.009

[28] Shah G H Panwar S K Chaturvedi P P Kane S N Isolated primary extranodal lymphoma of the oral cavity: A series of 15 cases and review of literature from a tertiary care cancer centre in India Indian J Med Paediatr Oncol 2011 32 02 76 81 22174494 10.4103/0971-5851.89776 PMC3237184

[29] Kemp S Gallagher G Kabani S Noonan V O'Hara C Oral non-Hodgkin's lymphoma: review of the literature and World Health Organization classification with reference to 40 cases Oral Surg Oral Med Oral Pathol Oral Radiol Endod 2008 105 02 194 201 17604660 10.1016/j.tripleo.2007.02.019

[30] Takahashi H Tezuka F Fujita S Okabe H Primary extranodal non-Hodgkin's malignant lymphoma of the oral region: analysis of 11 autopsy cases J Oral Pathol 1987 16 05 241 250 3116185 10.1111/j.1600-0714.1987.tb01487.x

[31] Triantafillidou K Dimitrakopoulos J Iordanidis F Gkagkalis A Extranodal non-Hodgkin lymphomas of the oral cavity and maxillofacial region: a clinical study of 58 cases and review of the literature J Oral Maxillofac Surg 2012 70 12 2776 2785 22494508 10.1016/j.joms.2012.01.018

[32] Eisenbud L Sciubba J Mir R Sachs S A Oral presentations in non-Hodgkin's lymphoma: a review of thirty-one cases. Part II. Fourteen cases arising in bone Oral Surg Oral Med Oral Pathol 1984 57 03 272 280 6584818 10.1016/0030-4220(84)90183-x

[33] Menter T Bodmer-Haecki A Dirnhofer S Tzankov A Evaluation of the diagnostic and prognostic value of PDL1 expression in Hodgkin and B-cell lymphomas Hum Pathol 2016 54 17 24 27045512 10.1016/j.humpath.2016.03.005

[34] Petroff M G Chen L Phillips T A Hunt J S B7 family molecules: novel immunomodulators at the maternal-fetal interface Placenta 2002 23(Suppl A):S95 S101 11978065 10.1053/plac.2002.0813

[35] Dong H Strome S E Salomao D R Tumor-associated B7-H1 promotes T-cell apoptosis: a potential mechanism of immune evasion Nat Med 2002 8 08 793 800 12091876 10.1038/nm730

[36] Azuma T Yao S Zhu G Flies A S Flies S J Chen L B7-H1 is a ubiquitous antiapoptotic receptor on cancer cells Blood 2008 111 07 3635 3643 18223165 10.1182/blood-2007-11-123141 PMC2275025

[37] Georgiou K Chen L Berglund M Genetic basis of PD-L1 overexpression in diffuse large B-cell lymphomas Blood 2016 127 24 3026 3034 27030389 10.1182/blood-2015-12-686550

[38] Nicolae A Pittaluga S Abdullah S EBV-positive large B-cell lymphomas in young patients: a nodal lymphoma with evidence for a tolerogenic immune environment Blood 2015 126 07 863 872 25999451 10.1182/blood-2015-02-630632 PMC4536540

[39] Chen B J Chapuy B Ouyang J PD-L1 expression is characteristic of a subset of aggressive B-cell lymphomas and virus-associated malignancies Clin Cancer Res 2013 19 13 3462 3473 23674495 10.1158/1078-0432.CCR-13-0855 PMC4102335

[40] Fest T Cerhan J R Gandhi M K Validation of elevated blood soluble PD-L1 as an independent prognostic marker in newly diagnosed diffuse large B-cell lymphoma (DLBCL) Blood 2014 124 2998

